# Accelerated amyloid fibril formation at the interface of liquid–liquid phase‐separated droplets by depletion interactions

**DOI:** 10.1002/pro.5163

**Published:** 2025-01-28

**Authors:** Keiichi Yamaguchi, Joji Mima, Kichitaro Nakajima, Hiroki Sakuta, Kenichi Yoshikawa, Yuji Goto

**Affiliations:** ^1^ Graduate School of Engineering Osaka University Osaka Japan; ^2^ Global Center for Medical Engineering and Informatics Osaka University Osaka Japan; ^3^ Faculty of Life and Medical Sciences Doshisha University Kyoto Japan; ^4^ Center for Complex Systems Biology Universal Biology Institute, The University of Tokyo Tokyo Japan

**Keywords:** liquid droplet, macromolecular crowding, supersaturation, α‐Synuclein, β_2_‐microglobulin

## Abstract

Amyloid fibril formation of α‐synuclein (αSN) is a hallmark of synucleinopathies. Although the previous studies have provided numerous insights into the molecular basis of αSN amyloid formation, it remains unclear how αSN self‐assembles into amyloid fibrils in vivo. Here, we show that αSN amyloid formation is accelerated in the presence of two macromolecular crowders, polyethylene glycol (PEG) (MW: ~10,000) and dextran (DEX) (MW: ~500,000), with a maximum at approximately 7% (w/v) PEG and 7% (w/v) DEX. Under these conditions, the two crowders induce a two‐phase separation of upper PEG and lower DEX phases with a small number of liquid droplets of DEX and PEG in PEG and DEX phases, respectively. Fluorescence microscope images revealed that the interfaces of DEX droplets in the upper PEG phase are the major sites of amyloid formation. We consider that the depletion interactions working in micro phase‐segregated state with DEX and PEG systems causes αSN condensation at the interface between solute PEG and DEX droplets, resulting in accelerated amyloid formation. Ultrasonication further accelerated the amyloid formation in both DEX and PEG phases, confirming the droplet‐dependent amyloid formation. Similar PEG/DEX‐dependent accelerated amyloid formation was observed for amyloid β peptide. In contrast, amyloid formation of β_2_‐microglobulin or hen egg white lysozyme with a native fold was suppressed in the PEG/DEX mixtures, suggesting that the depletion interactions work adversely depending on whether the protein is unfolded or folded.

## 1 INTRODUCTION

Many proteins form amyloid fibrils, ordered aggregates associated with serious amyloidosis (Benson et al., 2020; Chiti & Dobson, 2017). Now that the atomic structures of various amyloid fibrils have been shown to be ordered β‐sheet structures achieved by hydrophobic interactions, electrostatic interactions, and van der Waals interactions (Fitzpatrick et al., 2017; Iadanza et al., 2018; Radamaker et al., 2021; Sawaya et al., 2021; Scheres et al., 2023; Wilkinson et al., 2023), it is important to clarify the mechanisms underlying their formation. Amyloid fibrils are formed under various solvent conditions by distinct mechanisms (Furukawa et al., 2020; Goto et al., 2024) (Figure S1). These include: (i) a counter ion‐binding mechanism observed under acidic conditions in the presence of a moderate concentration of salts, (ii) a salting‐out mechanism observed under high‐salt conditions independent of the pH, (iii) a hydrophobic additive‐binding mechanism observed in the presence of moderate concentrations of alcohols, detergents like SDS, or the membrane surface, and (iv) pI‐precipitation under low‐salt conditions. Common to these mechanisms, a supersaturated state of responsible proteins and their breakdown are required to trigger crystal‐like amyloid formation (Goto et al., 2024; Goto, Noji, et al., 2022; Portugal Barron & Guo, 2023). Supersaturation plays a role in hemoglobin S fiber formation, the molecular basis of sickle cell anemia (Hofrichter et al., 1976), and also plays a role in various types of lithiasis (Gour & Gazit, 2021). Supersaturation will be an essential factor for comprehensive understanding of proteostasis (Balch et al., 2008; Goto et al., 2024).

On the other hand, macromolecular crowding under cellular conditions is important to understand amyloid formation in the context of proteostasis. The effects of molecular crowders on amyloid formation have been explained by three mechanisms (Minton, 2000, 2001; Nakajima et al., 2022; Nakano et al., 2014): (i) volume exclusion effects, increasing the effective concentration of amyloidogenic proteins, thus accelerating amyloid formation (Uversky et al., 2002); (ii) interactions with crowders (e.g., serum albumin), decelerating amyloid formation (Nakajima et al., 2022; Seeliger et al., 2013); and (iii) decrease in the diffusion constant, decelerating amyloid formation (Munishkina et al., 2004).

Another important topic related to amyloid formation under macromolecular crowding is liquid–liquid phase separation and resultant biomolecular condensates or droplets observed increasingly in disordered proteins. There are cases whereby amyloid formation is preceded by the liquid–liquid phase separation (Alberti & Hyman, 2021; Dec et al., 2024). For example, the low‐complexity domain of FUS protein formed phase‐separated droplets before the formation of amyloid fibrils (Murray et al., 2017). Importantly, Lipinski et al. (2022) showed that biomolecular condensates markedly speed up amyloid formation when proteins localize to their interface. More recently, Linsenmeier et al. (2023) also showed that amyloid formation does not occur homogeneously inside the droplets but is promoted at the interface of the condensates. On the other hand, Ray et al. (2023) reported nanoscale α‐synuclein (αSN) droplets in polyethylene glycol (PEG), where PEG does not partition inside the droplets and the PEG excluded αSN droplets maturate to amyloid fibrils.

To address the spaciotemporal evolution of amyloid formation, we should consider the depletion interactions driven by entropic effects (Marenduzzo, Finan, & Cook, 2006; Zosel et al., 2020), which might work in amyloid formation under macromolecular crowding and in vivo. A mixture of PEG and dextran (DEX) is known to exhibit liquid–liquid phase separation as well as classical two‐phase separation with upper PEG and lower DEX phases. Tange et al. (2021) showed that PEG/DEX mixtures accelerated amyloid formation of human recombinant prion protein. They suggested that the interface between PEG and DEX phases is the site of amyloid nucleation. Song et al. (2016) showed that the interface of DEX emulsion in the PEG‐rich phase absorbs amyloid fibrils of hen egg white lysozyme (HEWL), forming fibrillosomes stabilized by amyloid fibrils. Nakatani et al. (2018) studied the specific localization of monomeric actin (G‐actin), polymeric fibrillar actin (F‐actin), and bundled F‐actin in liquid–liquid phase separated PEG/DEX microdroplets. It should be noted that F‐actin formation is a supersaturation‐limited polymerization of G‐actin, although it is distinct from amyloid formation. Interestingly, G‐actin was distributed evenly between PEG and DEX phases, F‐actin was entrapped specifically within microdroplets rich in DEX, and bundled F‐actins localized at the interface, causing the deformation of DEX droplets. These suggest that the location of protein molecules in a PEG/DEX droplet system depends on the geometry of polymers in addition to the solvent properties. Nakatani et al. (2018) suggested the importance of depletion interactions in microphase separation on actin polymerization. Specific localization of microtubles and kinesin together with the generation of vortex flow has also been reported (Sakuta et al., 2023).

Here, we examined the effects of PEG (MW: ~10,000) and DEX (MW: ~500,000) mixtures on the amyloid fibril formation of αSN, an intrinsically disordered protein of 140 amino acid residues associated with several synucleinopathies, including Parkinson's disease (PD), dementia with Lewy bodies, and multiple‐system atrophy (Scheres et al., 2023). Amyloid formation of αSN was markedly accelerated in a mixture of PEG/DEX with an optimum at approximately 7% (wt/vol) PEG and 7% (wt/vol) DEX, where apparent two‐phase separation was established. When the PEG and DEX phases before amyloid formation were recovered and incubated, the PEG phase dominantly showed amyloid formation. Fluorescence microscope images confirmed that amyloid fibrils were located at the surface of DEX droplets in the PEG phase. Ultrasonication, known to trigger amyloid formation (Goto et al., 2024), further accelerated αSN amyloid formation in both PEG and DEX phases, probably by increasing the number of droplets. Similar PEG/DEX‐dependent acceleration of amyloid formation was observed for the Aβ1‐40 peptide. In contrast, the PEG/DEX mixture suppressed amyloid formation of β_2_‐microglobulin (β2m) (Hatano et al., 2021; Nakajima et al., 2022; Yamamoto & Gejyo, 2005) and HEWL (Nitani et al., 2017; Umemoto et al., 2014), proteins with a native fold. Taken together, PEG/DEX droplet‐dependent depletion interactions result in adverse outcomes in amyloid formation depending on the protein conformation, adding another key factor determining the solubility and supersaturation‐dependent amyloid formation.

## 2 RESULTS

### 2.1 Amyloid fibril formation in two‐phase separated PEG/DEX mixtures

**FIGURE 1:**
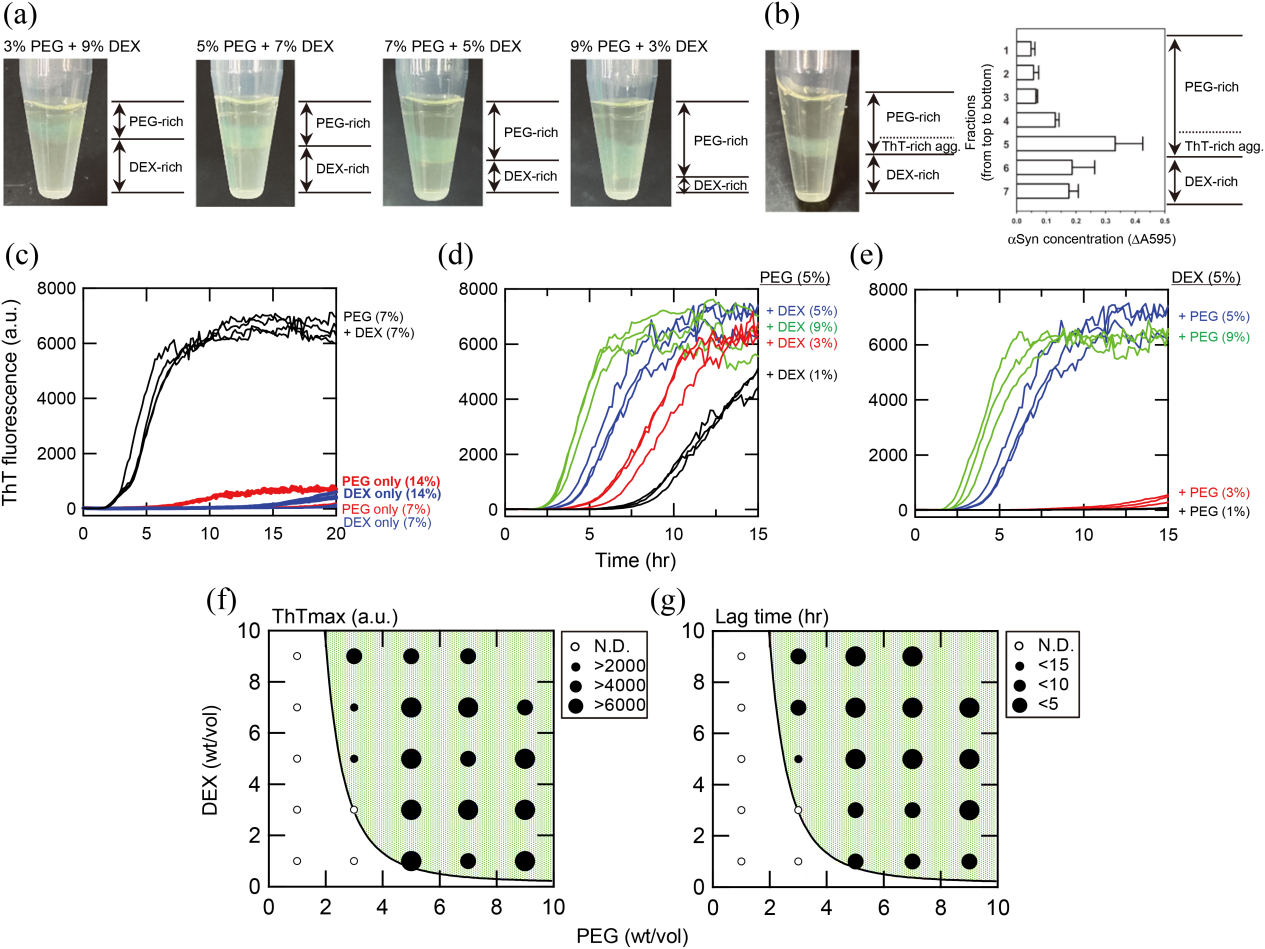
PEG‐ and DEX‐dependent acceleration of αSN amyloid fibril formation monitored by ThT fluorescence at pH 7.0. The αSN concentration was 30 μM. (a) Representative pictures of αSN amyloid formation in the presence of varying concentrations (3%, 5%, 7%, or 9% PEG) of PEG/DEX at a total of 12% (w/v). (b) Distribution of αSN in the PEG/DEX phases after amyloid formation. After the reaction with 7% PEG and 7% DEX, fractions at 50 μL each were collected and their αSN concentrations were assayed by the Bradford method using Protein Assay CBB Solution (Nacalai Tesque). Mean and S.D. values were determined from three independent reactions. (c) Both PEG and DEX were required for acceleration of αSN amyloid formation. (d, e) Both DEX (d) and PEG (e) contributed to accelerated amyloid formation when PEG (d) and DEX (e) were fixed at 5% (w/v). (f, g) PEG and DEX concentration‐dependent phase diagram of αSN amyloid formation monitored by the ThT fluorescence maximum (f) and lag time (g). The size of solid circles indicates the ThT maximum or lag time as defined in the panels. The PEG/DEX phase‐separated region is indicated by a green color.

Amyloid fibril formation of αSN was examined under varying concentrations of PEG and DEX, each between 1% and 9% (wt/vol) (Figure 1). Without PEG/DEX at pH 7.0, no amyloid formation was observed for 30 μM αSN within 20 h monitored by thioflavin T (ThT) fluorescence (Figure S2a). Neither PEG nor DEX alone induced amyloid formation (Figure 1c). On the other hand, typical kinetics of amyloid formation with a sigmoidal increase in ThT fluorescence were observed in the presence of PEG/DEX mixtures (Figure 1d,e), where PEG and DEX concentrations were higher than 3 and 1% (wt/vol), respectively (Figure 1f,g). The amount of amyloid fibrils estimated from ThT fluorescence was proportional to the αSN concentration (Figure S2a). As a control, β2m did not form amyloid fibrils at the quiescent conditions (Figure S2b). In the following quiescent experiments, we used αSN at 30 μM.

To make the PEG and DEX concentration‐dependent phase diagram of αSN amyloid formation, we plotted the ThT maximum values (Figure 1f) and lag times (Figure 1g) against the DEX/PEG concentrations. The increases in PEG or DEX concentrations both accelerated the amyloid formation, with a broad maximum at approximately 5%–9% (wt/vol) PEG and 3%–7% (wt/vol) DEX. Under the conditions of low concentrations of PEG/DEX or in the absence of either PEG or DEX, these additives were miscible with water. On the other hand, upon combined addition of PEG and DEX, two‐phase separation occurred within a few min after mixing and the boundary persisted during incubation. Interestingly, amyloid formation (closed circles) occurred in the region of two‐phase separation, as shown by the green color in the phase diagram (Figure 1f,g).

The volumes of PEG and DEX phases after phase separation depended on the concentrations of the two components: When the total of PEG and DEX concentrations was fixed at 12% (wt/vol), the boundary moved downward with a decrease in the DEX concentration (Figure 1a). After amyloid formation, a visible opal green color and ThT fluorescence were detected at the bottom of the upper PEG phase, although this does not indicate the site of formation. Instead, it is likely that preformed amyloid fibrils precipitated at the bottom of the PEG phase (Figure 1a). We noticed that the phase‐separated droplets are rather stable at the conditions of 7% (wt/vol) PEG and 7% (wt/vol) DEX compared with the composition closer to the bimodal boundary line, and thus hereafter we have adapted the 7% PEG/7% DEX as the standard solvent conditions.

**FIGURE 2:**
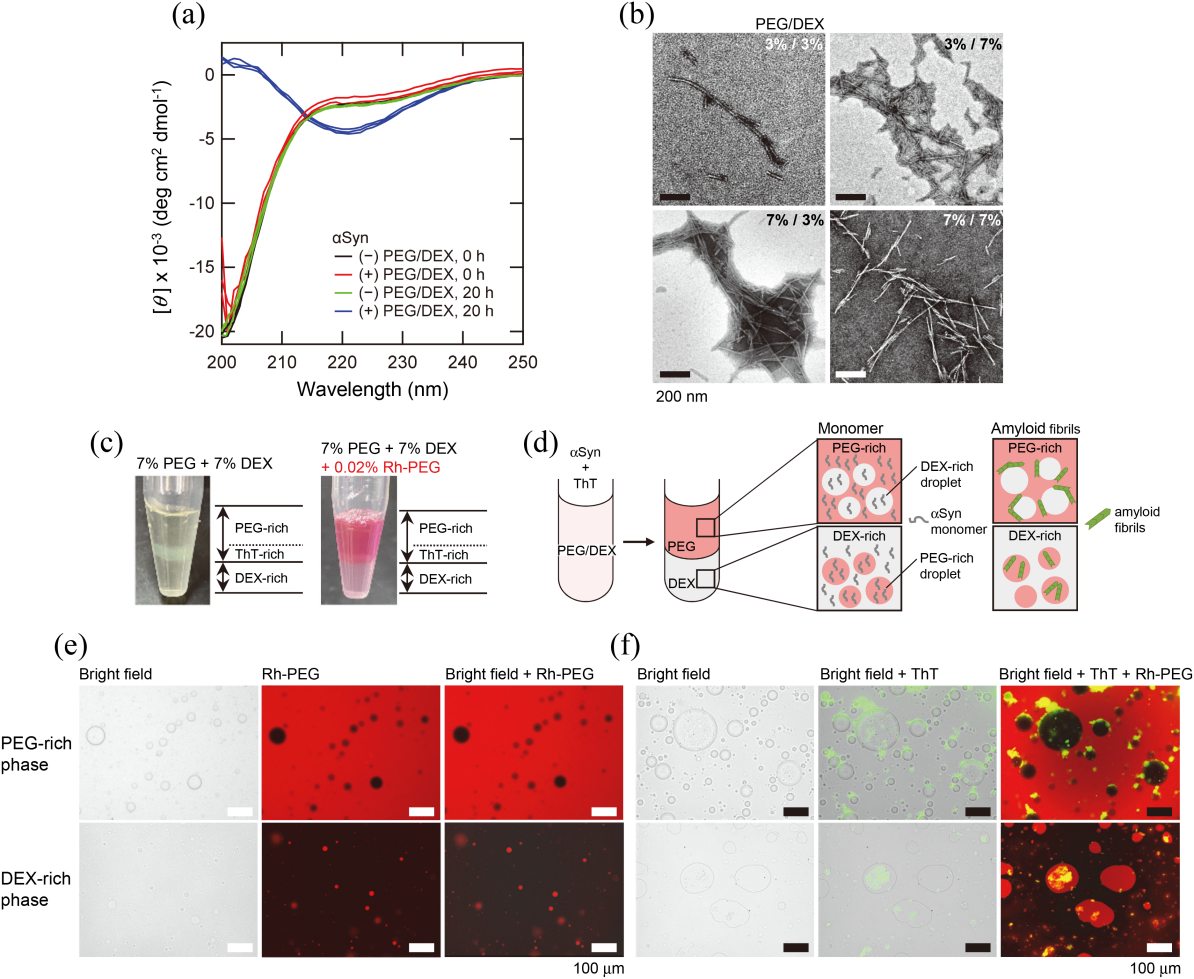
Characterization of amyloid fibrils of αSN formed in the PEG/DEX mixtures at pH 7.0 and 37°C. (a) Far‐UV CD spectra of αSN (30 μM) in the presence of PEG (9%) and DEX (1%) after incubation for 0 and 20 h at 37°C. For comparison, the spectra in the absence of PEG and DEX at 0 and 20 h are shown. (b) TEM images of αSN amyloid fibrils formed in varying concentrations of PEG and DEX after incubation for 20 h at 37°C. Scale bars are 200 nm. (c) Left: A photo of the two‐phase separated upper PEG layer, including the ThT‐specific layer and lower DEX layer. Right: The same two‐phase separation was prepared with a PEG/DEX mixture including 0.02% Rh‐PEG. (d) Schematic models of two‐phase separation of PEG and DEX, droplet formation, and amyloid fibril formation. Rhodamine‐PEG (red) and ThT (green) were used to specifically observe PEG and amyloid fibrils, respectively. (e, f) Fluorescence microscope images before and after amyloid fibril formation in the PEG‐rich (upper panels) and DEX‐rich (lower panels) phases. (e) Bright field (left), Rh‐PEG (middle) and their overlapping (right) images. (f) Bright field (left), overlapping images of bright field/ThT (middle) and bright field/ThT/Rh‐PEG (right) images. Scale bars are 100 μm.

The secondary structure and morphology of amyloid fibrils formed in the PEG/DEX mixtures were examined by far‐UV circular dichroism (CD) (Figure 2a) and transmission electron microscopy (TEM) (Figure 2b), respectively, showing typical characteristics of αSN amyloid fibrils, as we previously reported (Furukawa et al., 2020; Sawada et al., 2020). Although the disease‐dependent differences in atomic structure and morphology are currently important topics (Fitzpatrick et al., 2017; Radamaker et al., 2021; Sawaya et al., 2021; Wilkinson et al., 2023), our methodologies did not generate such details.

We also examined the pH‐ and NaCl concentration‐dependent amyloid formation of αSN monitored by ThT fluorescence at 7% (wt/vol) PEG and 7% (wt/vol) DEX (Figure S3). The pH‐dependence at 100 mM NaCl showed an optimum at pH 6.0–7.0 (Figure S3a,b), consistent with our previous paper (Furukawa et al., 2020). The NaCl concentration‐dependence at pH 7.0 in 100 mM sodium phosphate buffer showed the decrease in the maximum ThT value without changing the lag time between 0 and 0.2 M NaCl (Figure S3d,e). Fluorescence microscopy showed ThT‐positive αSN amyloid clusters located outside of droplet‐like structures obtained by bright‐field mode (Figure S3).

### 2.2 Amyloid fibril formation on the surface of DEX droplets in the PEG phase

**FIGURE 3:**
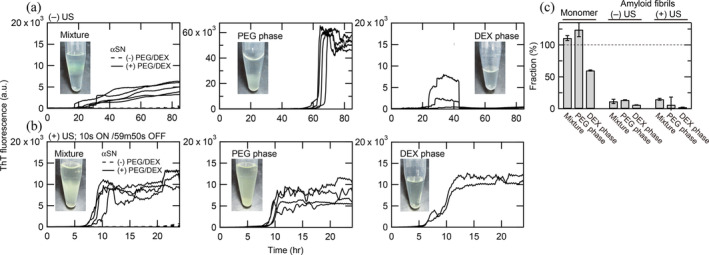
Amyloid fibril formation of 30 μM αSN in two‐phase separated PEG/DEX mixtures at pH 7.0 and 37°C. (a, b) Kinetics of amyloid formation monitored by ThT fluorescence in the PEG/DEX mixture (left), PEG phase (middle), and DEX phase (right) under quiescence (a) or ultrasonication (b). Insets show pictures of αSN solutions after amyloid formation. (c) Distribution of αSN in different phases estimated from elution profiles.

It is known that, even after two‐phase separation, small numbers of DEX and PEG droplets exist in separated PEG and DEX phases, respectively. Using rhodamine‐labeled PEG and fluorescence microscopy, we monitored PEG and DEX droplets in the two‐phase separated PEG/DEX mixtures (Figure 2c,d). Before the formation of amyloid fibrils, both PEG and DEX droplets were observed in the DEX and PEG phases, respectively (Figure 2e). After the formation of amyloid fibrils, ThT fluorescence spots were identified on the surfaces of DEX droplets in the PEG phase (Figure 2f, top), and were identified inside the PEG droplets in the DEX phase (Figure 2f, bottom). The ThT fluorescence spots were also observed outside the surfaces of droplets in the PEG phase. We estimated that the quantity ratio of the ThT fluorescence around and outside of the surface of the droplets was ~6:4. Because some ThT fractions appear to be connected to or extended from the aggregate surfaces, it was difficult to distinguish these ThT fractions exactly. Because the separated PEG phase markedly contributed to the total ThT fluorescence of the PEG/DEX mixtures (Figure 3a), the results confirmed that surfaces of DEX droplets in the PEG phase are the major sites of amyloid formation “under quiescence”.

The lag time (~20 h) in Figure 3a is longer than that in Figure 1c (~3 h). This was likely due to a change in the batch of DEX. Because the DEX batch (Pharmacia Fine Chemicals, Sweden) used for producing Figure 1 was not available in the following experiments, we used a new batch (Pharmacosmos, Denmark) for the subsequent experiments. Importantly, the lag times differed between the two DEX batches, although the ThT maximum values are almost identical between them: Comparing the amyloid formation at 7% PEG and 7% DEX in Figures 1c and 3a. Because αSN is highly hydrophilic, it takes a long time to form amyloid fibrils under the quiescent conditions (Yagi et al., 2005, 2015b). It is considered that the amyloid formation is markedly accelerated in the PEG/DEX mixtures and the degree of acceleration depends on DEX batches.

### 2.3 Amyloid fibril formation in separated PEG/DEX phases

To identify the site of amyloid fibril formation, three fractions (upper, middle including boundary, and lower phases) at 7% (wt/vol) PEG and 7% (wt/vol) DEX were recovered after the completion of two‐phase separation, but before amyloid formation. Then, amyloid formation of each fraction and the mixture were monitored by ThT fluorescence (Figure 3a). Amyloid formation occurred dominantly in the PEG phase. Thus, although amyloid deposits were observed at the bottom of the upper PEG phase at the end of the reaction (Figure 1a), amyloid formation in fact occurred at the separated PEG phase and amyloid fibrils precipitated at the boundary of PEG/DEX phases.

Concentrations of αSN in the PEG and DEX phases were determined by reversed‐phase HPLC (Figures 3c and S4). Before the formation of amyloid fibrils, the concentration ratio at the upper PEG and lower DEX phases was 67:33. After the formation of amyloid fibrils, the concentrations of soluble αSN were estimated by applying the solutions to HPLC after centrifugation. αSN concentrations of both phases decreased, representing an equilibrium of low solubilities after breakdown of supersaturation.

### 2.4 Effects of ultrasonication

Ultrasonic irradiation is a useful agitation to accelerate amyloid fibril formation by forced breakdown of supersaturation (Goto et al., 2024; Goto, Nakajima, et al., 2022; Nakajima et al., 2016, 2022). Ultrasonic irradiation is also effective for fragmenting particles including amyloid fibrils based on the effects of cavitation‐induced mass movement and concomitant shear forces (Chatani et al., 2009). These effects are useful for preparing seeds for seed‐dependent amyloid fibril propagation (Naiki et al., 1997). We expected ultrasonic irradiation of PEG/DEX mixtures to agitate the two‐phase separation, producing a larger number of droplets. This would increase the surface of droplets, leading to the further acceleration of amyloid formation.

We used the HANABI system (Goto, Nakajima, et al., 2022; Nakajima et al., 2021; Umemoto et al., 2014) to apply ultrasonic irradiation efficiently. In the absence of PEG/DEX mixtures, no amyloid formation was observed within 24 h. Upon ultrasonic irradiation in the presence of PEG/DEX mixtures, amyloid formation occurred with a lag time of ~8 h (Figure 3b). When the PEG and DEX phases were recovered soon after the two‐phase separation and ultrasonicated separately, both phases showed cooperative amyloid formation with lag times of ~8 and ~6 h for the PEG and DEX phases, respectively. Maximal ThT fluorescence intensities for the DEX phase were slightly larger than those for the PEG phase. Because viscosity of the DEX phase was high, the results indicate that suppression of amyloid formation in the DEX phase (Figure 3a) was due to the difficulty of solution mixing in the DEX phase under quiescence. It is likely that the ultrasonic irradiation caused efficient mixing of the DEX phase, producing a larger number of droplets, and leading to enhanced amyloid formation.

### 2.5 Generality of PEG/DEX micro phase‐separation

**FIGURE 4:**
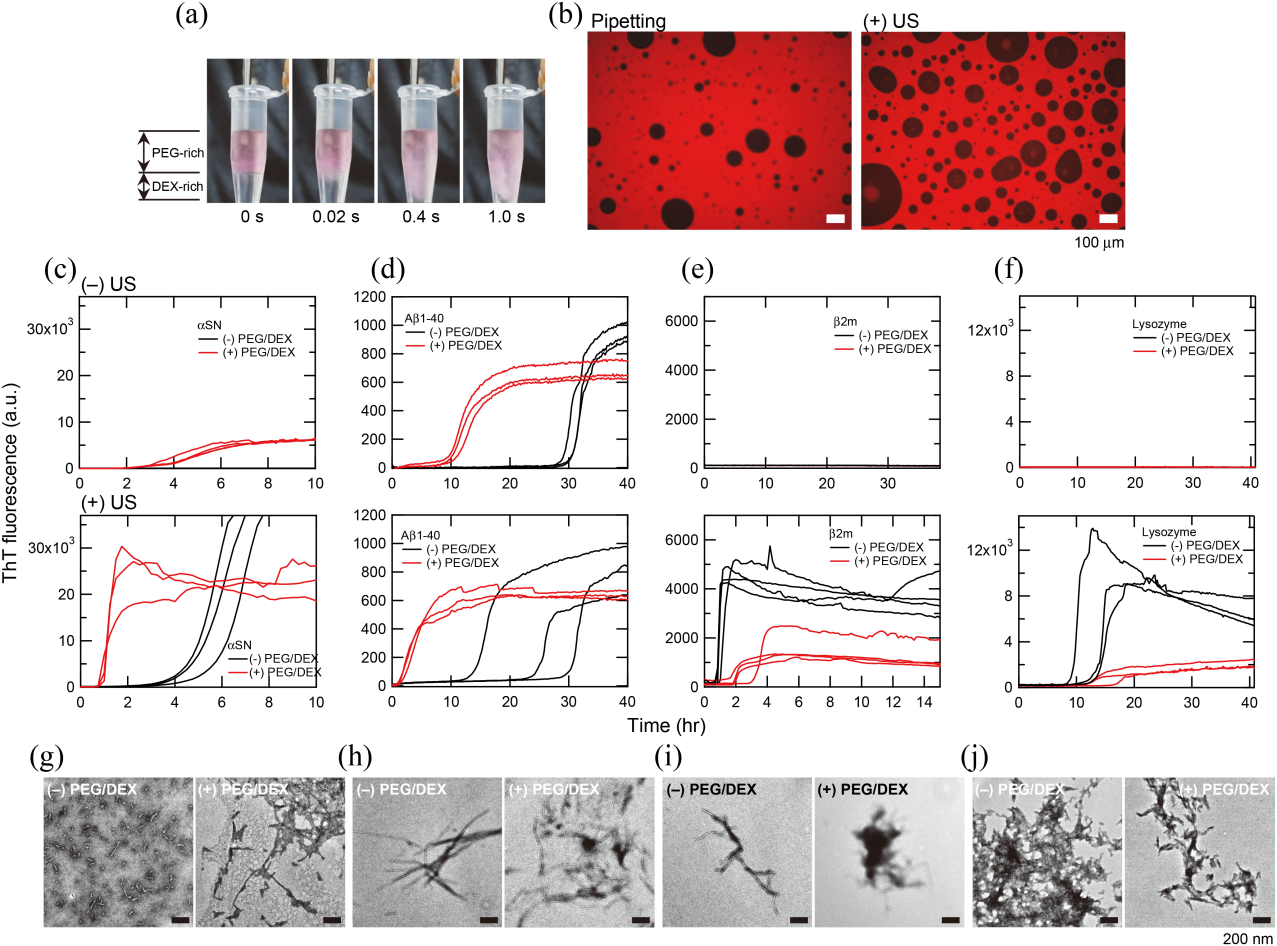
Effects of ultrasonication on the amyloid formation of various proteins in the presence or absence of PEG/DEX mixtures. (a) Ultrasonication‐dependent rapid agitation of the two‐phase separated PEG/DEX mixtures monitored by Rh‐labeled PEG. A hone‐type ultrasonicator was used. (b) Fluorescence microscopy images of DEX droplets visualized with Rh‐labeled PEG. Droplets were prepared by pipetting (left) or ultrasonication (right). (c–f) The HANABI 2000 system was used to monitor the amyloid formation of αSN (c), Aβ(1–40) (d), β2m (e) and HEWL (f) without ultrasonication (top) or with ultrasonication (bottom). The amyloid formation of αSN in 7% PEG and 7% DEX without ultrasonication are reproduced from Figure 1c. (g–j) TEM images of amyloid fibrils of αSN (g), Aβ(1–40) (h), β2m (i), and HEWL (j) prepared in the absence (left) or presence (right) of the PEG/DEX mixtures under the ultrasonication. Scale bars are 200 nm.

To examine the generality of the PEG/DEX‐dependent acceleration of amyloid fibril formation, we used Aβ1‐40, β2m, and HEWL (Figure 4). Aβ1‐40 and β2m are associated with Alzheimer's disease and dialysis‐related amyloidosis, respectively (Benson et al., 2020; Chiti & Dobson, 2017; Yamamoto & Gejyo, 2005). HEWL is a model amyloidogenic protein (Nitani et al., 2017; Umemoto et al., 2014). We used the HANABI 2000 system, the newest version with increased synchronization and sensitivity (Nakajima et al., 2021). Because αSN quickly formed amyloid fibrils under the ultrasonication, the concentration of αSN was adjusted to be 10 μM for the HANABI 2000 assay. The HANABI system accelerated amyloid formation both in the presence and absence of PEG/DEX mixtures, and the reaction in the presence of PEG/DEX mixtures was much faster.

Under quiescence, Aβ1‐40 formed amyloid fibrils cooperatively with a lag time of approximately 30 h (Figure 4d). The addition of PEG/DEX mixtures shortened the lag time to 10 h. When solutions were ultrasonicated with HANABI 2000, the reactions in the presence of PEG/DEX mixtures were further accelerated, occurring without a lag time. Ultrasonication also accelerated the reactions in the absence of PEG/DEX mixtures, although the degree of synchronization was low.

β2m taking a native fold at pH 7 did not form amyloid fibrils under quiescence at 60°C in the presence or absence of PEG/DEX mixtures (Figure 4e, see also Noji et al., 2021; Noji et al., 2019). However, the HANABI 2000 system forced amyloid formation with a lag time of approximately 1 h (Figure 4c). Contrary to our expectation, the addition of PEG/DEX mixtures notably suppressed the reaction in terms of lag time and ThT fluorescence (Figure 4e). Similar suppression of PEG/DEX‐dependent amyloid formation was observed for HEWL in the presence of 0.5 M GuHCl at pH 2. Without ultrasonication, HEWL with a native fold formed no amyloid fibrils regardless of the presence or absence of PEG/DEX mixtures. Ultrasonication enabled amyloid formation under both conditions. However, both the lag time and maximal ThT fluorescence indicated the PEG/DEX‐dependent suppression of amyloid formation. These results showed that the effects of PEG/DEX take place adversely depending on whether the protein is folded or unfolded.

## 3 DISCUSSION

### 3.1 Depletion interaction‐dependent local condensation and amyloid fibril formation

The basis of depletion interactions between two large particles in a solution of small particles is that the small particles cannot penetrate the large particles, which leads to a loss of configurational entropy of small particles near the surface of the large ones (Asakura & Oosawa, 1954; Marenduzzo, Finan, & Cook, 2006; Vrij, 1976; Zosel et al., 2020). Then, the two large particles associate to increase the volume available to the small particles (increasing their entropy). Alternatively, when the two large particles approach one another, the small ones are excluded from the volume between the two, thereby exerting an unopposed bombard force from small ones equivalent to the osmotic pressure to keep them together (Marenduzzo, Finan, & Cook, 2006; Marenduzzo, Micheletti, & Cook, 2006). The depletion effects that cause the interaction between proteins are particularly important in a crowded solution and are suggested to cause the collapse of intrinsically disordered proteins in the presence of crowding agents (Kang et al., 2015; Zosel et al., 2020). Related effects caused by translational entropy of water is also essential in biological self‐assembly processes like protein folding and amyloid formation (Kinoshita, 2009, 2013).

The contribution of depletion effects to the free energy of protein interactions has often been regarded as minor compared with other interactions (i.e., electrostatic interactions, van der Waals interactions, and hydrophobic interactions): The contribution might be approximately 1 *k*_B_ *T*, comparable with the free energy associated with one hydrogen bond in a protein (Marenduzzo, Finan, & Cook, 2006; Marenduzzo, Micheletti, & Cook, 2006). However, it is expected that the depletion effects become important under crowding conditions in living systems; for example, cytoplasmic solution contains 30–40 weight % of macromolecules, including skeletal proteins, RNA, DNA, etc. (Ellis, 2001; Kuznetsova et al., 2014; Minton, 2000, 2001; Zhou et al., 2008; Zimmerman & Trach, 1991). Actually, there are suggestions that they play a role in driving various cellular events including amyloid fibril formation and sickle cell hemoglobin polymerization, both supersaturation‐dependent phenomena (Marenduzzo, Finan, & Cook, 2006; Marenduzzo, Micheletti, & Cook, 2006). On the other hand, the biomolecular condensates, e.g. the presynaptic condensates where αSN natively resides, are also shown to accumulate the charge at their interfaces (Hoffmann et al., 2023), and the charge accumulation may accelerate chemical reactions such as redox reactions (Dai et al., 2023) or amyloid fibril formation (Shen et al., 2023).

**FIGURE 5:**
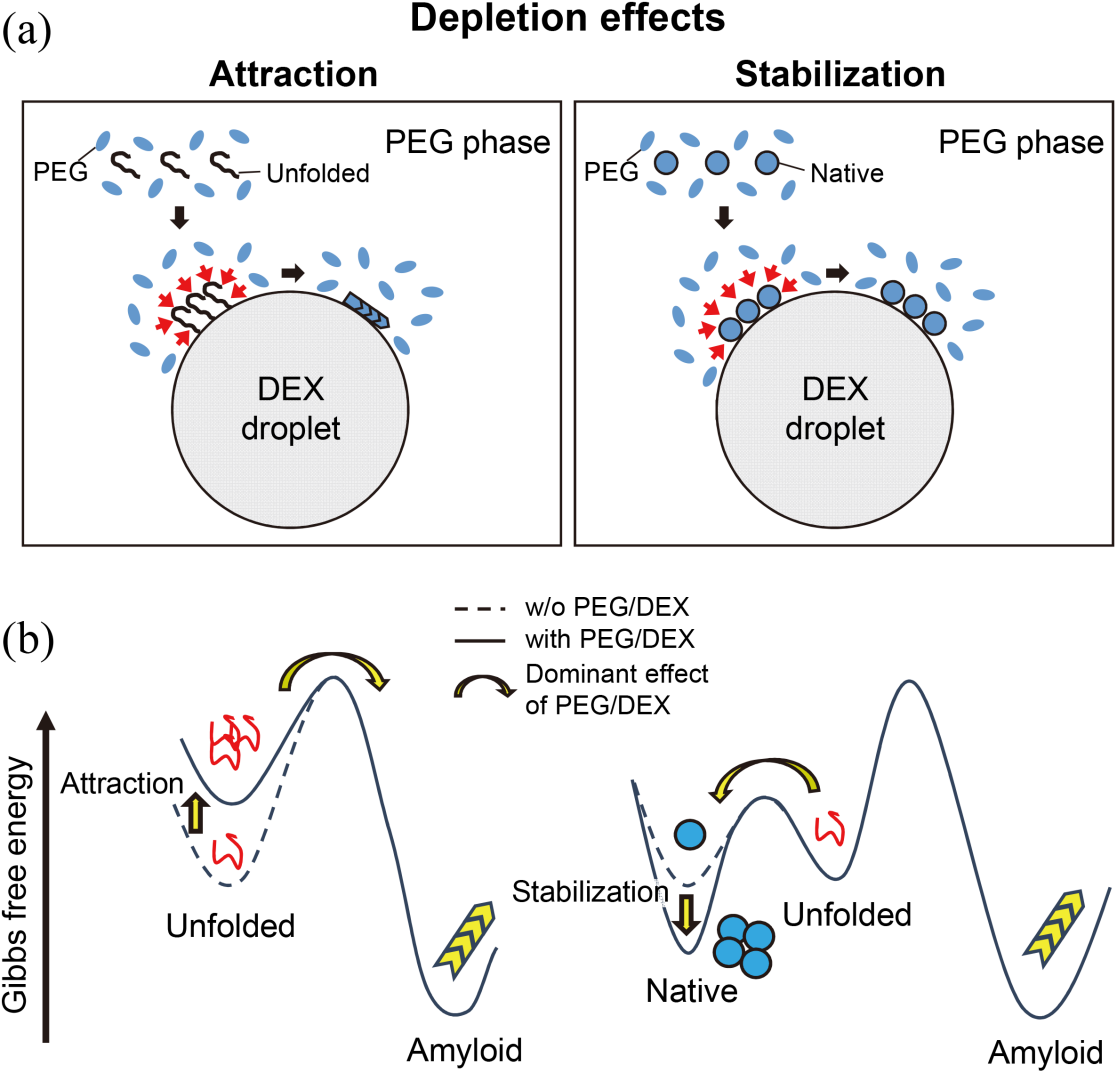
Models of depletion effects on amyloid formation. (a) Depletion interactions concentrate amyloidogenic proteins on the surface of DEX droplets in the PEG phase, leading to accelerated amyloid formation or suppressed amyloid formation for the unfolded and folded proteins, respectively. (b) Modulations of Gibbs free energy of amyloid formation by depletion interactions.

Here, the average molecular weights of αSN, PEG and DEX were 14,500, ~10,000, and ~500,000, respectively. The partition of αSN between PEG and DEX phases before the formation of amyloid fibrils was 67:33 (Figure 3c), suggesting no specific interaction between αSN and PEG or DEX. It is expected that, when the size of the assemblies (αSN) among polymers (DEX) becomes larger than the void space in DEX droplets, the assemblies tend to be localized near the interface of the PEG and DEX phases (Nakatani et al., 2018; Nishio et al., 2021; Sakuta et al., 2023; Wilkinson et al., 2023). Then, it is likely that depletion interactions between PEG and DEX molecules worked effectively to concentrate αSN at the surface of DEX droplets in the PEG phase (Figure 5a), modulating Gibbs free energy of amyloid formation (Figure 5b). The opposite situation is likely to happen on the inside of PEG droplets in the DEX phase. An increase in the local concentration increases the degree of supersaturation, thus increasing the propensity for amyloid formation (Goto et al., 2024; Nakajima et al., 2022). Although water molecules are often considered as small molecules in depletion interactions and can also contribute to the observed PEG/DEX effects, we do not consider its role because water is miscible with both PEG and DEX phases.

Song et al. (2016) showed that the interface of DEX emulsion in the PEG‐rich phase absorbs amyloid fibrils of HEWL, forming fibrillosomes stabilized by amyloid fibrils. Fibrillosomes were proposed to be useful for creating self‐standing protein capsules. The specific localization of amyloid fibrils at the interface was explained by the absorption energy and interface tension. However, the specific localization of HEWL amyloid fibrils at the interface of DEX emulsion is similar to those observed here. Lipinski et al. (2022) showed that biomolecular condensates markedly speed up αSN amyloid formation when αSN molecules localize to their interface. They also observed that condensates suppress amyloid formation by sequestering and stabilizing amyloidogenic proteins, thereby providing living cells with a possible protection mechanism against amyloid formation. Recently, Linsenmeier et al. (2023) reported that amyloid formation of low‐complexity domain of hnRNPA does not occur homogeneously inside the droplets but is promoted at the interface of the condensates. These papers suggest that the interface specific localization of amyloid fibrils is caused by the interface‐specific affinity or binding. However, it is likely that the interface localization is attributed to depletion interactions, which is a simple geometrical factor contributing to spaciotemporal evolution of amyloid formation.

### 3.2 Outcome of depletion effects depending on protein conformation

We expected the PEG/DEX‐dependent acceleration of amyloid formation to be common to varying amyloidogenic proteins. In fact, Tange et al. (2021) reported that liquid–liquid phase separation of full‐length prion protein in a PEG/DEX system accelerated amyloid fibril formation of prion protein, although they did not identify the site of formation. As for Aβ1‐40, we observed notable acceleration of amyloid fibril formation in the presence of PEG/DEX mixtures under both quiescence and ultrasonication (Figure 4d).

Contrary to this, suppression was observed for the folded β2m and HEWL (Figure 4e,f). These results indicate that the acceleration or deceleration of amyloid formation depends on the protein conformation. As the magnitude of attractive interaction owing to the depletion effect is expected to be sensitively dependent on the change in effective volume or stiffness/flexibility of macromolecules, condensation of a native state and its stabilization will be preferred under the crowding conditions (Minton, 2000, 2001; Nakano et al., 2014; Nishio et al., 2021; Vasilevskaya et al., 1995).

### 3.3 Unifying various mechanisms of amyloid fibril formation

Although supersaturation and its breakdown are required processes for amyloid fibril formation and the crystallization of solutes (Goto et al., 2024; Goto, Noji, et al., 2022), there are various ways to reach supersaturation (Figure S1). Furukawa et al. (2020) classified mainly solvent conditions which decrease the solubility of “denatured” amyloid precursor proteins. Those conditions then bring about supersaturation and moreover increase the degree of supersaturation (i.e., increasing the propensity for amyloid formation (Goto et al., 2024; Nakajima et al., 2022)). Alternatively, the degree of supersaturation can be elevated by increasing the protein concentration: here, depletion interactions play a role. Spontaneous amyloid formation starts by breaking supersaturation through additional unknown factors.

The effects of varying conditions accelerating amyloid formation can be classified into solvation (or chemical) and geometrical (or physical) ones, both increasing the degree of supersaturation (Figure S1). Among them, the salting‐out effects caused by Hofmeister salts have both aspects because the available solvent volume decreases due to water‐hydrated ion molecules (Goto et al., 1990; Timasheff & Arakawa, 1988). In conclusion, we added depletion interactions to a list of various mechanisms producing amyloid fibrils, providing a comprehensive view of amyloid fibril formation. We also showed that depletion interactions can play adverse roles in amyloid fibril formation depending on the protein conformation. Considering depletion interactions will further advance our understanding of the mechanism of amyloid formation and therapeutic strategies against amyloidosis.

## 4 MATERIALS AND METHODS

### 4.1 Reagents

Recombinant human αSN was expressed in *Escherichia coli* and purified as described previously (Yagi et al., 2005). Recombinant human β2m with an additional methionine residue at the N terminus was expressed using *Escherichia coli* and purified as described previously (Chiba et al., 2003). HEWL (Nitani et al., 2017) and Aβ1–40 (Yagi et al., 2015a) were purchased from Nacalai Tesque (Kyoto, Japan) and Peptide Institute Inc. (Osaka, Japan) and were used as described previously. The fluorescence dye ThT was obtained from Wako Pure Chemical Industries (Osaka, Japan). PEG with an average molecular weight of 7400–10,200 was from Nacalai Tesque. Fluorescence‐labeled Rh‐PEG with an average molecular weight of 5000 was from Nanocs (New York, USA). DEX with an average molecular weight of 450,000–550,000 was from Pharmacia Fine Chemicals (Uppsala, Sweden) and Pharmacosmos (Holbaek, Denmark). All the other reagents were purchased from Nacalai Tesque.

### 4.2 Kinetic ThT assay for amyloid formation

αSN solutions were mixed with PEG and DEX in 100 mM sodium phosphate buffer at pH 7.0, containing 25 μM ThT and 100 mM NaCl. The reaction mixtures were transferred into a black 96‐well plate (100 μL/well; Greiner 675076) sealed with a transparent plastic film (Greiner 676070) and immediately assayed for amyloid formation by monitoring the ThT fluorescence (λex = 450 nm, λem = 490 nm) at 37°C for 15 or 20 h (with 5‐ or 10‐min intervals) using an MTP‐810 microplate reader (Corona Electric, Tokyo, Japan). To quantitatively compare the amyloid formation, the maximum ThT fluorescence values and lag time, defined as the time at which ThT fluorescence reached 1/10th of the maximum, are summarized in Figures 1 and S3.

Using the HANABI 2000, similar experiments were performed under ultrasonication with 10 μM αSN, 10 μM Aβ1‐40, 42 μM β2m, and 70 μM HEWL at 7% (w/v) PEG and 7% (w/v) DEX. Buffer conditions were αSN: 100 mM sodium phosphate at pH 7.0, 100 mM NaCl, 25 μM ThT, Aβ1‐40: 20 mM sodium phosphate at pH 7.0, 50 mM NaCl, 10 μM ThT, β2m: 20 mM sodium phosphate pH 7.0, 500 mM NaCl, 10 μM ThT, and HEWL: 10 mM HCl pH ~2, 0.5 M GuHCl, 10 μM ThT. The HANABI 2000 system optimized for accelerated amyloid formation was used (Nakajima et al., 2021, 2022). The reaction mixtures were added to a 96‐well plate (200 μL/well; 675096, Greiner) sealed with a plastic film (Greiner 676070) and immediately assayed for amyloid formation by monitoring the ThT fluorescence (λex = 450 nm, λem = 490 nm) at 37°C for αSN, Aβ1–40 and HEWL, and 60°C for β2m. The sample solutions inside the plate were irradiated with ultrasound at a frequency of ~30 kHz, an optimized frequency for accelerating amyloid formation (Nakajima et al., 2021, 2022). During the experiments, ultrasonic irradiation was performed with duty cycles comprising 0.3‐s irradiation and 30‐s quiescence incubation. ThT fluorescence was measured using SF6 software (Version 5.12.1, Corona Electric Co., Ltd.).

### 4.3 Fluorescence microscopy

Fluorescence microscopy of ThT‐positive αSN amyloid fibrils in PEG/DEX mixtures was performed using a BZ‐X710 fluorescence microscope (Keyence, Osaka, Japan) equipped with a Plan Fluor 10X NA 0.30 objective lens (Nikon) and GFP‐B and TRITC/PE fluorescence filters (Keyence). After incubating αSN (30 μM) in the presence of PEG (7%), DEX (7%), and Rh‐PEG (0.02%, where indicated) at 37°C for 20 h, as in the kinetic ThT assays, a drop (10 μL each) of the αSN reactions harvested from the PEG‐rich phases was placed on a microscope slide (S1112, Matsunami Glass), covered with an 18‐mm coverslip (Matsunami Glass), and subsequently subjected to fluorescence microscopy. The bright‐field, ThT‐fluorescence, and Rh‐fluorescence images obtained were processed using the BZ‐X viewer application (Keyence) and ImageJ2 software (National Institutes of Health).

### 4.4 CD spectroscopy

Far‐UV CD spectra of αSN amyloid fibrils in PEG/DEX mixtures were measured at 25°C using a J‐820 spectropolarimeter (Jasco, Tokyo, Japan) and a 1‐mm pathlength quartz cell with a scan rate of 20 nm/min. Before CD measurements, αSN (30 μM) was incubated at 37°C for 20 h (at pH 7.0) as in the kinetic ThT assays, in the presence of 9% PEG and 1% DEX at pH 7.0. For the negative controls, the αSN solutions without the 20‐h incubation were also subjected to CD spectroscopy in the presence or absence of PEG/DEX. After subtracting the spectrum of the protein‐free solutions, the CD spectra obtained at 200–250 nm were expressed as the mean residue ellipticity [*θ*] (deg cm^2^ dmol^−1^).

### 4.5 Negative‐stain electron microscopy

Transmission electron microscopy (TEM) of amyloid fibrils was performed using a Hitachi H‐7650 transmission electron microscope operating at an acceleration voltage of 80 kV with a magnification of ×15,000, as described previously (Furukawa et al., 2020; Sawada et al., 2020; Yamaguchi et al., 2021). Amyloid fibrils from the PEG‐rich phase were diluted with a 4‐fold volume of deionized water. Aliquots of the diluted αSN suspensions (5 μL each) were placed on copper grids (400‐mesh) covered with carbon‐coated collodion film (Nissin EM), incubated (room temperature, 2 min), negatively stained with 1.5% phosphotungstic acid (5 μL each, 1 min), dried (room temperature, more than 16 h), and then subjected to TEM analyses.

### 4.6 Estimation of soluble fractions

To evaluate the concentrations of soluble αSN monomers in PEG/DEX mixtures, separated PEG and DEX phases before amyloid formation were applied to reversed‐phase COSMOSIL 5C_4_‐AR‐300 column chromatography (Nacalai Tesque) using the HPLC system (Gilson). To evaluate the concentrations of soluble αSN monomers after the amyloid formation under the quiescent and ultrasonic conditions, the supernatants after the centrifugation at 100,000 ***g*** at 20°C for 1 h were applied to reversed‐phase COSMOSIL 5C_4_‐AR‐300 column chromatography. The samples were eluted with a gradient of 25%–40% acetonitrile/H_2_O including 0.05% TFA. The mean and S.D. values obtained were determined from at least three independent reactions.

## AUTHOR CONTRIBUTIONS

**Keiichi Yamaguchi:** Conceptualization; investigation; funding acquisition; writing – review and editing; validation; formal analysis; methodology. **Joji Mima:** Investigation; conceptualization; writing – review and editing. **Kichitaro Nakajima:** Conceptualization; investigation; writing – review and editing; funding acquisition. **Hiroki Sakuta:** Conceptualization; writing – review and editing. **Kenichi Yoshikawa:** Conceptualization; writing – review and editing; supervision. **Yuji Goto:** Conceptualization; supervision; writing – original draft; project administration; funding acquisition.

## CONFLICT OF INTEREST STATEMENT

The authors declare no conflict of interest.

## Supporting information


Data S1.
