# Supplementary material for: Accelerated amyloid fibril formation at the interface of liquid–liquid phase‐separated droplets by depletion interactions
Source: Protein Sci. 2025 Jan 28;34(2):e5163. doi: 10.1002/pro.5163 (PMC11774873; doi:10.1002/pro.5163)
Supplement: Supplementary file 1 — Data S1. [file PRO-34-e5163-s001.pdf]

Supporting Information for

Accelerated amyloid fibril formation at the interface of liquid-liquid phase-separated droplets by depletion interactions

Keiichi Yamaguchi, Joji Mima, Kichitaro Nakajima, Hiroki Sakuta, Kenichi Yoshikawa, and Yuji Goto

Yuji Goto
Email: gtyj8126@protein.osaka-u.ac.jp

This PDF file includes:

Supporting Figures S1 to S4

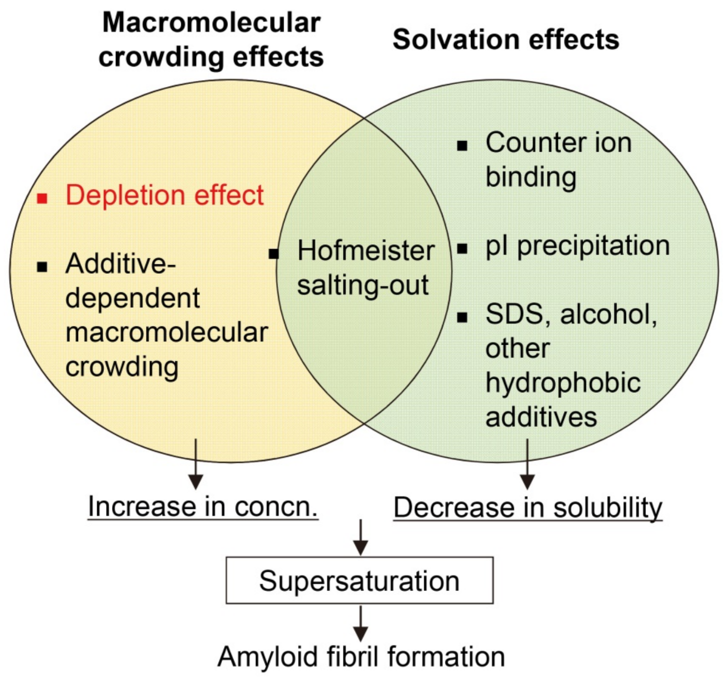

Figure S1 Various conditions inducing amyloid fibrils are classified into macromolecular crowding and solvation effects. Both effects induce supersaturation of unfolded precursor proteins, leading to amyloid formation after breaking the supersaturation. The same mechanism may work adversely on the folded proteins, stabilizing the native sate and possibly native state crystallization.

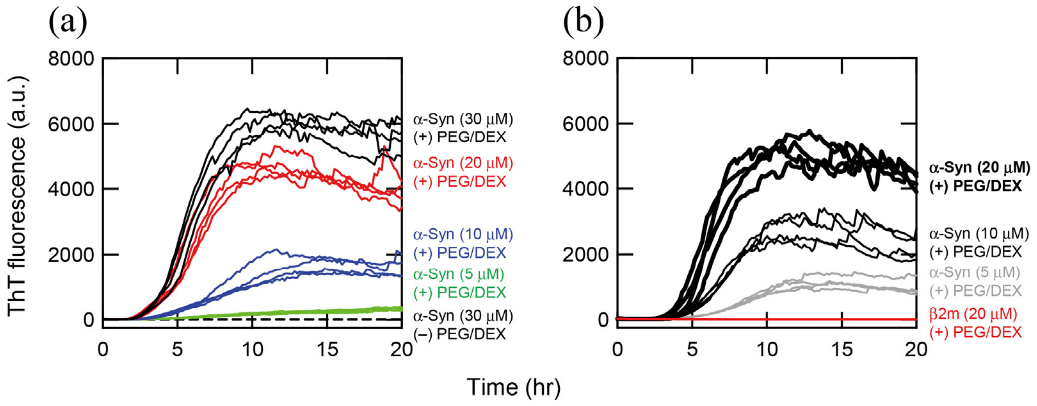

Figure S2 (a) The rate and amount of αSN amyloid formation at 7% PEG and 7% DEX were proportional to the αSN concentration. (b) b2m (20 μM) formed no amyloid fibrils under the same conditions as αSN formed amyloid fibrils. At least four independent reactions were analyzed under the quiescent conditions.

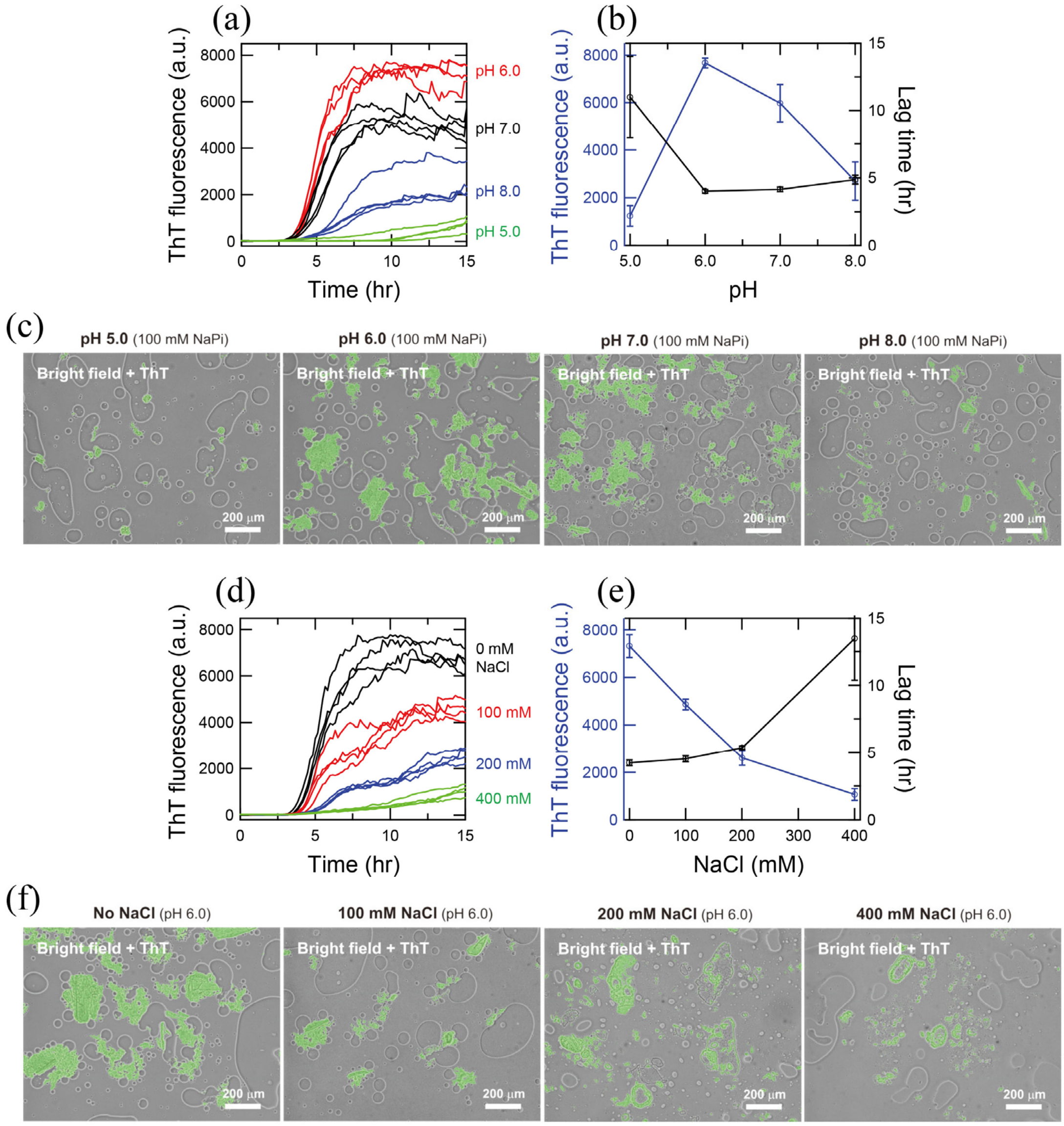

Figure S3 Effects of pH (a-c) and NaCl concentration (d-f) on the αSN amyloid formation in PEG/DEX mixtures. (a, d) Kinetics of amyloid formation under varying pH (a) and NaCl concentrations (d). αSN (30 μM) was incubated with PEG/DEX (7% each) and assayed by measuring ThT fluorescence, as in Figure 1, but at various pH or NaCl concentrations. (b, e) Dependences on pH (b) and NaCl concentration at pH 7.0 (e) of maximum ThT fluorescence (left axis) and lag time (right axis). At least four independent reactions were analyzed. Error bars indicate S.D. (c, f) After the reactions, solutions were subjected to fluorescence microscopy, obtaining bright-field and ThT-fluorescence images. Scale bars, 200 μm.

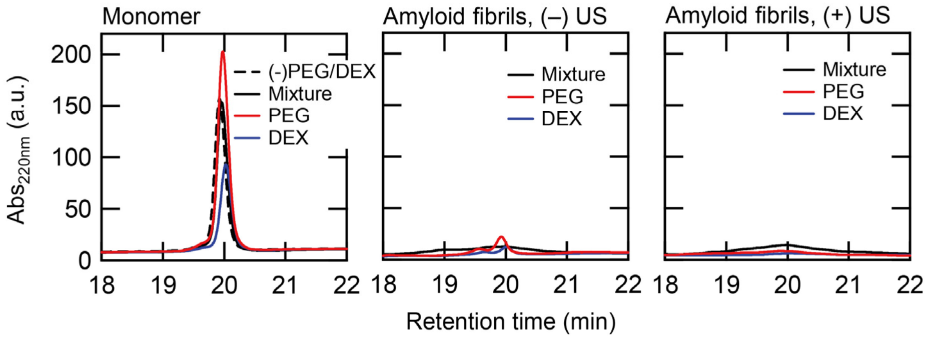

Figure S4 Partition of αSN in PEG and DEX phases assayed by reverse-phase HPLC. Elution profiles of αSN in the PEG and DEX phases before (left) and after amyloid formation under the quiescent (middle) and ultrasonic (right) conditions.
